# ICM experimental is growing, from bench via bedside to big data—and back!

**DOI:** 10.1186/s40635-023-00507-5

**Published:** 2023-04-11

**Authors:** Nicole P. Juffermans

**Affiliations:** 1 Department of Intensive Care, OLVG Hospital, Amsterdam, The Netherlands; 2 Laboratory of Translational Intensive Care, Erasmus Medical Center, Rotterdam, The Netherlands

Two complementary journals serve the European Society of Intensive Care Medicine (ESICM) community and their impact is growing. *Intensive Care Medicine (ICM)* is the highest-ranking journal dealing exclusively with intensive care research. *ICM experimental (ICMx)* will receive an impact factor in 2023—and this impact factor will apply to all papers published in *ICMx* as from 2007, when indexing started. While *ICM* focuses on publishing clinical studies, *ICMx* aims to improve understanding of the underlying pathophysiology of critical care syndromes to advance discovery science [1]. Both sides of research are essential for our patients [1–3].

Randomized trials and the systematic analysis of pooled results are regarded as superior in ranking evidence and are the backbone of guideline development [4]. Randomized trials, meta-analyses and guidelines all impact the way we treat patients on a daily basis. However, to advance our care, improved understanding of the diseases and syndromes of our critically ill patients is paramount. We need better diagnostics, better biomarkers, better monitoring. Most of all, we need therapies.

Translating fundamental discoveries to the development of therapeutic targets involves multiple steps. Traditionally, translational research was viewed as a path stretching '*from bench to bedside’*, along insights derived from in vitro to animal models to clinical studies. In the recent years, however, we are recognizing that the complexity of many critical care syndromes require attempts to develop a precision medicine approach, using strategies that group patients into more homogeneous cohorts, with shared biological features (phenotypes). In the end, the aim is to define ‘treatable traits’, which are groups of patients with a specific pathophysiology that respond to a specific therapy [5]. This knowledge can feed into clinical trial design, randomizing only patients with a specific trait, with higher success rates while minimizing patient exposure.

*ICMx* aims to be the platform for all types of research that can contribute to a step in the process of discoveries with a potential to improve trial design in ICU. Data science, which is the field of study dedicated to the principled extraction of knowledge from complex data, is particularly relevant in the critical care setting because of the complexity of critical illness combined with the availability of large amounts of digital data. Machine learning can be employed to improve prediction based on observations, whereas data mining (e.g., of data generated by clinical trials) discovers novel insights within the data. *ICMx* considers such data science methodology also an important part of translational research (Fig. 1).

**Fig. 1:**
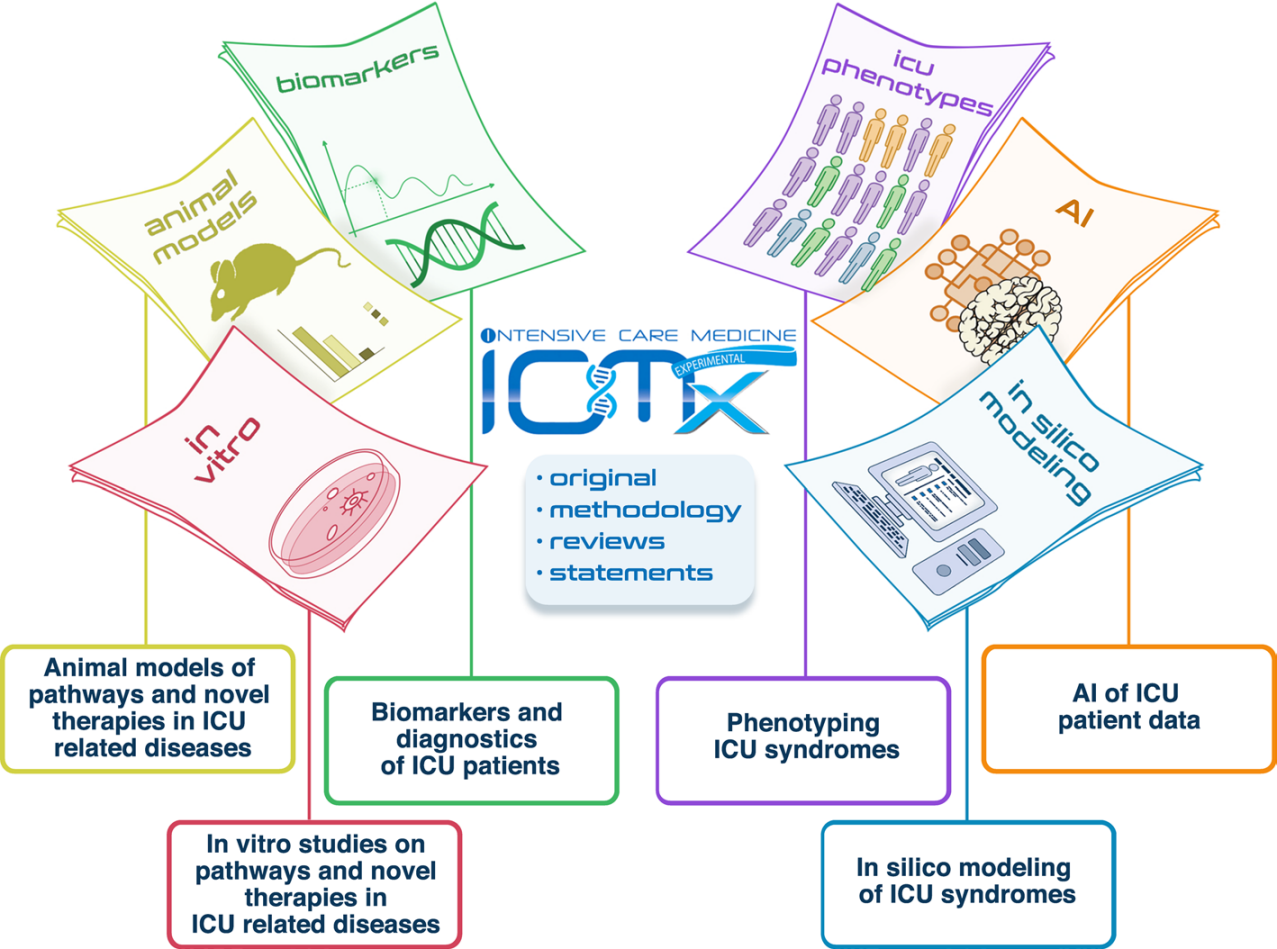
Types of translational research

Regarding the scope of *ICMx*, another important issue has to be pointed out. Experimental work is being done in many fields that have relevance for critical care, e.g., in the field of cardiology, in the field of metabolism, and in the field of pulmonology. Publication of such work is currently scattered across journals related to other specialties. *ICMx* aims to be a platform for studies that are performed in the intersection of ICU with these other specialties. In addition, *ICMx* recognizes that critical care medicine is not confined by ICU walls and that patients with acute derangements of physiology are also cared for in operating rooms and emergency departments.

To improve awareness of readers and researchers about this broad scope of *ICMx*, the editorial board has been re-structured. Sections on data science, perioperative medicine, cardiovascular medicine and metabolism have been added to already existing sections of sepsis, respiratory failure, neuro-IC and animal research. Thereby, *ICMx* aims to serve all researchers and ICU health care professionals committed to discovering better ways of caring for the acutely ill.

On behalf of the *ICMx* editorial team, I thank you for your continuous support and interest in *ICMx*. Our aim is to grow and to become a leading journal dedicated to translational ICU medicine. We propose it is time for an adjustment of the discovery pathway: *from bench *via* bedside to big data—and back!*

## Data Availability

Not applicable.
